# CTHRC1 Derived From Cancer‐Associated Fibroblasts Promotes Pancreatic Cancer Progression and Metastasis via the LIF‐STAT3 Pathway

**DOI:** 10.1002/cam4.71126

**Published:** 2025-08-02

**Authors:** Hang Yin, Yue Pan, Zhuang Li, Yong Liu, Jiatong Chen, Xin Chen, Chengfei Zhang, Feng Zhu, Chunzhao Yu

**Affiliations:** ^1^ Nanjing Medical University Nanjing China; ^2^ Department of General Surgery Nantong Hospital of Traditional Chinese Medicine, Nantong Hospital Affiliated to Nanjing University of Chinese Medicine Nantong China; ^3^ Department of General Surgery Sir Run Run Hospital, Nanjing Medical University Nanjing China; ^4^ Department of General Surgery Jianghan University Affiliated Hospital (Wuhan Sixth Hospital) Wuhan China

**Keywords:** cancer‐associated fibroblast, CTHRC1, pancreatic cancer, STAT3 signaling pathway, tumor microenvironment

## Abstract

**Background:**

Collagen triple helix repeat containing 1 (CTHRC1) is a secreted protein involved in tissue remodeling and fibrotic processes, which also suggests emerging roles in cancer. Studies have shown that it is mainly expressed in the outer membrane fibroblasts of injured arteries and in the neointimal smooth muscle cells, where it promotes cell migration and tissue damage repair. However, the regulatory role of CTHRC1 as a tumor microenvironment factor in pancreatic cancer is not well understood.

**Methods:**

We employed multi‐omics analysis combined with cellular and animal experiments to examine the association between CTHRC1 and the LIF/STAT3 pathway in pancreatic cancer clinical specimens. Using AsPC‐1/PANC‐1 and other cell lines, we conducted proliferation, migration, invasion, and signaling pathway studies, and elucidated the regulatory mechanism of CTHRC1 through genetic interventions and STAT3 inhibitors.

**Results:**

In this study, we found that CTHRC1 is highly expressed in the cancer‐associated fibroblasts (CAFs) of pancreatic cancer and is associated with poor prognosis in patients. Functionally, we observed that CTHRC1 in CAFs promotes the proliferation, migration, and invasion of pancreatic cancer cells both in vitro and in vivo. In mechanistic studies, RNA sequencing revealed that CTHRC1 promotes the proliferation and migration of pancreatic cancer cells through the LIF‐mediated STAT3 axis.

**Conclusion:**

These findings reveal the role of CTHRC1 in CAFs in pancreatic cancer, suggesting that it is an attractive therapeutic target and tumor marker. This study uncovers the biological mechanism of CTHRC1 in CAFs in pancreatic cancer, providing new strategies for the treatment of pancreatic cancer.

## 1 Introduction

Pancreatic cancer ranks as the seventh leading cause of global cancer deaths [1], with a 5‐year survival rate of merely 11% even in developed countries [2]. Notably, China demonstrates higher incidence and mortality rates that progressively increase annually, alongside a trend toward younger onset [3]. Pancreatic ductal adenocarcinoma (PDAC) constitutes over 90% of pancreatic malignancies [4]. Due to its insidious early symptoms, only 10%–15% of patients qualify for surgical resection, with high postoperative recurrence rates yielding a 5‐year survival rate of just 15%–25% [5]. This underscores the urgent need to explore PDAC‐specific mechanisms for novel diagnostic and therapeutic targets.

The tumor microenvironment (TME) functions as a dynamic adaptive structure that drives cancer progression by regulating metabolic reprogramming, immune evasion, and chemoresistance [6, 7]. A hallmark of PDAC is its highly heterogeneous TME, comprising diverse components including pancreatic cancer cells, fibroblasts, pancreatic stellate cells, tumor‐associated macrophages, immune cells, adipocytes, extracellular matrix (ECM), growth factors, cytokines, and exosomes [8, 9]. Cancer‐associated fibroblasts (CAFs), as core constituents of the PDAC TME, have been established as critical drivers of cancer progression. They promote tumor cell proliferation, angiogenesis, and pre‐metastatic niche formation through matrix remodeling and paracrine signaling [10, 11, 12, 13]. Although CAFs are known to interact with cancer cells through multiple pathways [10], significant knowledge gaps persist regarding their precise molecular mechanisms.

This study focuses on the secretory protein collagen triple helix repeat containing 1 (CTHRC1)—initially identified in injured arteries and found overexpressed in multiple cancers (e.g., liver and colorectal cancers) with poor prognostic associations [14, 15, 16]. We first discovered that CTHRC1 is significantly upregulated in PDAC‐associated CAFs and positively correlates with adverse patient outcomes. Through genetically engineered CAF models, our in vitro and in vivo experiments demonstrate CTHRC1's role in promoting PDAC proliferation, migration, and invasion. Furthermore, we elucidate that this effect is mediated via the leukemia inhibitory factor (LIF)/STAT3 axis, thereby establishing CTHRC1 as a potential therapeutic target for PDAC.

## 2 Materials and Methods

### 2.1 Reagents

Anti‐CTHRC1 antibody, anti‐α‐SMA antibody, anti‐Vimentin antibody, anti‐E‐cadherin antibody, anti‐α‐Tubulin antibody, anti‐GAPDH antibody, goat anti‐rabbit IgG (H + L) HRP, and goat anti‐mouse IgG (H + L) HRP secondary antibodies were purchased from Proteintech. Anti‐LIF antibody, STAT3, and phospho‐STAT3 (Tyr705) were obtained from ABclonal. LIF inhibitor EC330 was purchased from MedChemExpress (MCE).

### 2.2 Plasmids and siRNAs

pLKO.1‐shScramble and pLKO.1‐shCTHRC1 vectors were obtained from Sigma‐Aldrich, pLenti‐CMV‐GFP/puro and pLenti‐CMV‐CTHRC1 vectors were obtained from Miaoling Biotechnology, shCTHRC1‐1 targeted the human CTHRC1 mRNA sequences F (5′‐CCGGCCCATTGAAGCTATAATTTATCTCGAGATAAATTATAGC‐TTCAATGGGTTTTTG‐3′), R (5′‐AATTCAAAAACCCATTGAAGCTATAATTTAT‐CTCGAGATAAATTATAGCTTCAATGGG‐3′). shCTHRC1‐2 targeted the human CTHRC1 mRNA sequences F (5′‐CCGGCGGAGTGTACATTTACAAAGACTCGA‐GTCTTTGTAAATGTACACTCCGTTTTTG‐3′), R (5′‐AATTCAAAAACGGAGTG‐TACATTTACAAAGACTCGAGTCTTTGTAAATGTACACTCCG‐3′). All the siRNA were purchased from GenePharma, siCTHRC1‐1 targeted the human CTHRC1 mRNA sequences F (5′‐CCCAUUGAAGCUAUAAUUUTT‐3′), R (5′‐AAAUUAUAGCUUC‐AAUGGGTT‐3′) and siCTHRC1‐2 targeted the human CTHRC1 mRNA sequences F (5′‐CGGAGUGUACAUUUACAAATT‐3′), R (5′‐UUUGUAAAUGUACACUCCG‐TT‐3′).

### 2.3 Preparation and Standardization of CAF‐Conditioned Medium (CAFs‐CM)

To obtain CAF‐conditioned medium (CAFs‐CM), primarily cultured CAFs were seeded in iCell primary medium supplemented with 10% fetal bovine serum. Upon reaching 80%–90% confluence, cells were washed three times with sterile PBS and incubated with freshly prepared medium of identical specifications for 48 h. The collected supernatant was centrifuged at 1000 rpm for 5 min and filtered through 0.22‐μm membranes, with the filtrate serving as CAF‐CM for subsequent cytological experiments. CAF‐CM was either used immediately or stored at −80°C.

### 2.4 RNA Isolation and Quantitative Real‐Time PCR (qRT‐PCR)

Total RNA from cultured cell lines was extracted with an RNA extraction kit obtained from Vazyme and was reverse transcribed into cDNA with an RNA reverse transcription kit obtained from Vazyme. The qRT‐PCR analysis was performed with the AceQ q‐PCR SYBR Green Master Mix kit obtained from Vazyme. The $2^{-\Delta \Delta C_{t}}$ method was used to analyze the relative levels of the target gene. All primer sequences for the qRT‐PCR assay were listed in Table 1.

**TABLE 1:** List of primer sequences used for quantitative real‐time PCR.

Gene	Forward (5′–3′)	Reverse (5′–3′)	Species
CTHRC1	TGGACACCCAACTACAAGCA	GAACAAGTGCCAACCCAGAT	Human
LIF	CCCTGGTCCCTACTCAACAA	CTGGACCCTGACACCCTAAA	Human
GAPDH	ACCCAGAAGACTGTGGATGG	TTCAGCTCAGGGATGACCTT	Human

### 2.5 CCK‐8 Assay

Pancreatic cancer cells were seeded in 96‐well plates at a density of 2 × 10^3^ cells per well to set up a blank control group. After the cells adhered to the surface, 10 μL of CCK‐8 solution (from APExBIO) was added to each well and incubated at 37°C for 1–2 h. The absorbance of each well was then measured at 450 nm using a microplate reader. The same procedure was repeated on Days 1, 3, and 5, and the absorbance of each well was measured. The data was then analyzed to assess the proliferative ability of the pancreatic cancer cells.

### 2.6 EdU Proliferation Assay

The 5‐ethynyl‐2′‐deoxyuridine (EdU) assay was performed using the EdU Proliferation Assay Kit from Beyotime. Cells were seeded in confocal dishes at a density of 2 × 10^5^ cells per well and cultured for 24 h, followed by incubation with EdU for 3 h. The remaining steps were carried out according to the reagent instructions. After staining with DAPI, images were captured using a fluorescence microscope.

### 2.7 Colony Formation Assay

Pancreatic cancer cells were seeded in 6‐well plates at 1 × 10^3^ cells/well and cultured continuously for 7–14 days, with medium replenished as needed. When the majority of monoclonal colonies exceeded 50 cells, cultures were processed by aspirating medium, rinsing twice with 1× PBS, fixing with 4% paraformaldehyde for 20 min, staining with 0.1% crystal violet for 30 min, washing away excess dye with water, air‐drying, photographing colonies, and performing quantitative analysis and data processing using ImageJ software.

### 2.8 Transwell Assay

Cell migration and invasion assays were performed using 8‐μm pore Transwell chambers (Yeasen) as follows: (1) Migration: Serum‐starved pancreatic cancer cells (3–5 × 10^4^cells/well) were seeded in upper chambers, with lower chambers containing 500 μL experimental CAFs‐CM or control shNC/con CAFs‐CM (same‐batch preparations). (2) Invasion: Upper chambers were pre‐coated with 35 μL Matrigel (Matrigel:serum‐free medium = 1:8), avoiding bubble formation. After 1‐h solidification at 37°C, the migration protocol was repeated. Following 48‐h incubation, nonmigrated cells were removed from upper chambers. Cells were fixed with 4% paraformaldehyde for 20 min, stained with 0.1% crystal violet for 30 min, and washed to remove excess dye. Migrated cells were imaged under microscopy, with four random fields counted per group using ImageJ software.

### 2.9 Wound Healing Assay

Uniformly seeded pancreatic cancer cells in 6‐well plates were scratched at 80%–90% confluence using 200‐μL sterile pipette tips. After PBS washes, the medium was replaced with experimental CAFs‐CM or control shNC/con CAFs‐CM. Wound closure was monitored at standardized positions under fixed focal length and illumination parameters, with images captured at 12, 24, 36, and 48 h for quantitative analysis.

### 2.10 Animal Experiments

NCG mice were randomly divided into two groups: shNC group and shCTHRC1‐2 group, with 10 mice in each group. CAFs of shNC or shCTHRC1‐2 were resuspended with Aspc‐1 at a cell number of 1:3 in 100‐μL sterile PBS (5 × 10^5^ CAFs cells/mouse, 1.5 × 10^6^ Aspc‐1 cells/mouse) and injected from the tail of the pancreas of the mice; leakage of the cell suspension should be avoided during the injection. The mice were imaged in vivo after 8 weeks, after which the mice were euthanized and the pancreatic tumors were dissected out. The intestinal and liver tissues were dissected and photographed, and the number of metastatic lesions was recorded. The weight of the tumor was weighed after dissection, and the long and wide diameters of the tumor were taken by vernier caliper. The volume of the tumor was calculated using the formula *V* (mm^3^) = length × width^2^/2. The NCG mice used in this experiment were all 6‐week‐old male mice obtained from the Medical Laboratory Animal Center of Nanjing Medical University, housed under specific pathogen‐free conditions. All animal experiments were approved by the Animal Welfare Ethics Committee of Nanjing Medical University.

### 2.11 RNA‐Sequence

The global gene expression profiles of CTHRC1‐knockdown and control CAFs cells were examined by RNA‐seq in LC‐Bio, and then the Enrichr tools were used to analyze the biological processes.

### 2.12 Statistical Analysis

Statistical analyses were performed using GraphPad Prism 8.0 software. Data are presented as mean ± standard deviation. Parametric tests were selected based on experimental requirements: direct comparisons used Student's *t*‐test; multigroup comparisons employed one‐way ANOVA; nonparametric tests were applied when data violated normality assumptions. Statistical significance was defined as *p* < 0.05 (*), *p* < 0.01 (**), and *p* < 0.001 (***).

## 3 Results

### 3.1 CTHRC1 Is Highly Expressed in CAFs of Pancreatic Cancer and Is Associated With Poor Prognosis of Patients

**FIGURE 1:**
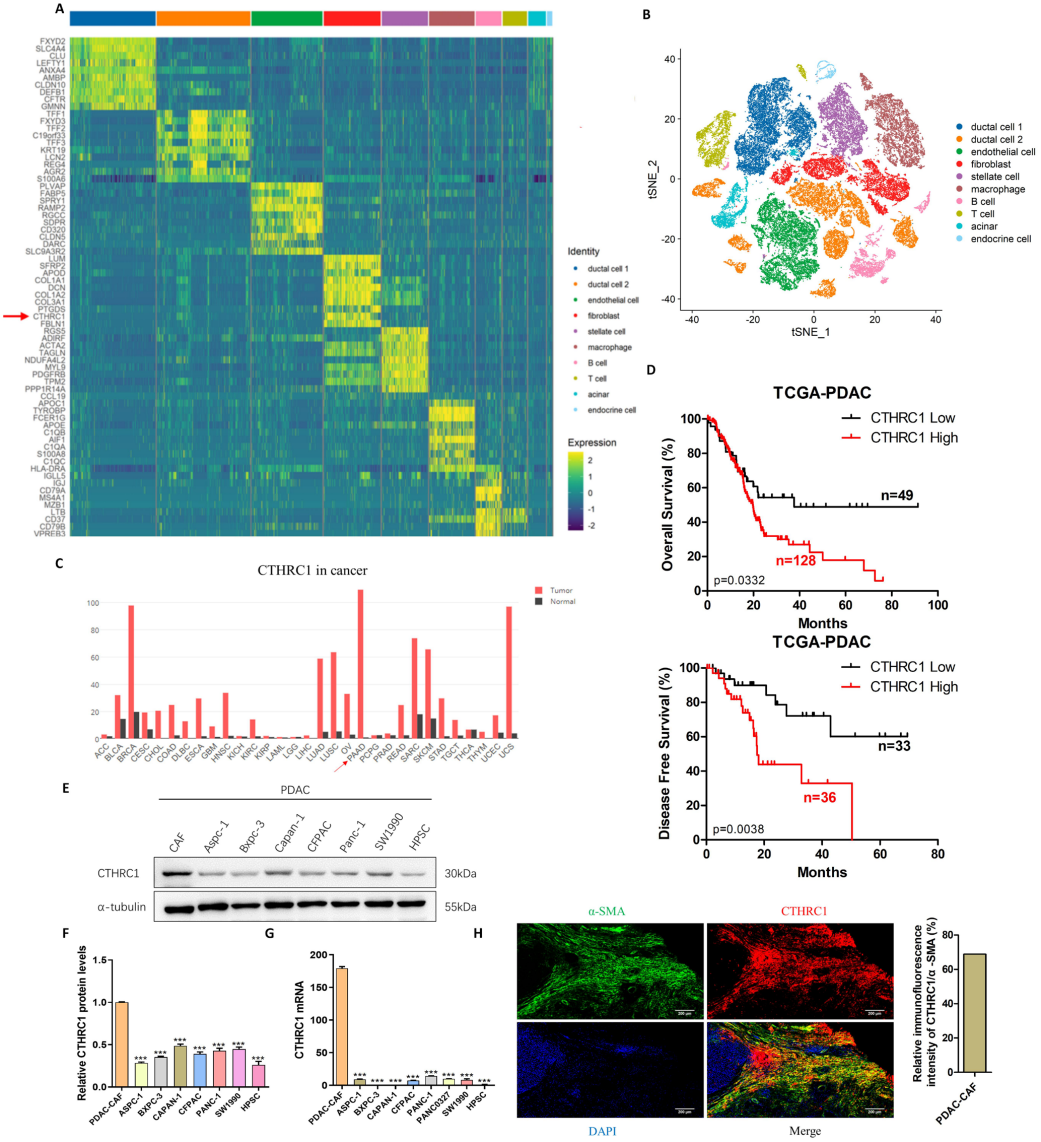
CTHRC1 is highly expressed in CAFs of pancreatic cancer and is associated with poor prognosis of patients. (A, B) Integrated single‐cell analysis combining quantitative heatmap (A) and t‐SNE clustering (B) demonstrated CTHRC1‐specific enrichment in pancreatic cancer CAFs. (C) GEPIA database results showed that CTHRC1 was highly expressed in pancreatic cancer tissues. (D) The overall survival and disease‐free survival of patients with high CTHRC1 expression were significantly worse than those with low CTHRC1 expression. (E) The expression of CTHRC1 in CAFs, pancreatic cancer cells, and pancreatic stellate cells was detected by Western Blot. (F) Protein gray scale analysis of CTHRC1 in CAFs, pancreatic cancer cells, and pancreatic stellate cells. (G) The expression of CTHRC1 in CAFs, pancreatic cancer cells, and pancreatic stellate cells was detected by q‐PCR. (H) CTHRC1 was colocalized with CAFs in pancreatic cancer (200 μm). ***p < 0.001.

To further explore the specific role of CAFs in pancreatic cancer and its important molecules, we first analyzed the single‐cell sequencing results [17] by bioinformatics analysis and found that CTHRC1 was significantly overexpressed in CAFs of pancreatic cancer, which was significantly higher than its expression level in pancreatic cancer cells and other cells (Figure 1A,B). Second, our analysis results from TCGA and GEPIA databases showed that CTHRC1 was significantly overexpressed in pancreatic cancer, which was significantly different from other tumors. Moreover, patients with high CTHRC1 expression had a significantly worse prognosis and higher recurrence and metastasis rates than those with low CTHRC1 expression (Figure 1C,D), suggesting that CTHRC1 may play an important role in the development of pancreatic cancer. Western Blot and q‐PCR further confirmed that CTHRC1 was highly expressed in CAFs, which was significantly higher than that in pancreatic cancer cells and pancreatic stellate cells (Figure 1E–G). Double‐labeled immunofluorescence staining of human pancreatic cancer tissues showed that CTHRC1 was significantly colocalized with CAFs in pancreatic cancer tissues (Figure 1H). Therefore, the above results suggest that CTHRC1 in CAFs may be one of the important molecules that play an essential role in promoting pancreatic cancer development.

### 3.2 CTHRC1 in CAFs Promotes Proliferation and Self‐Renewal of Pancreatic Cancer Cells In Vitro

**FIGURE 2:**
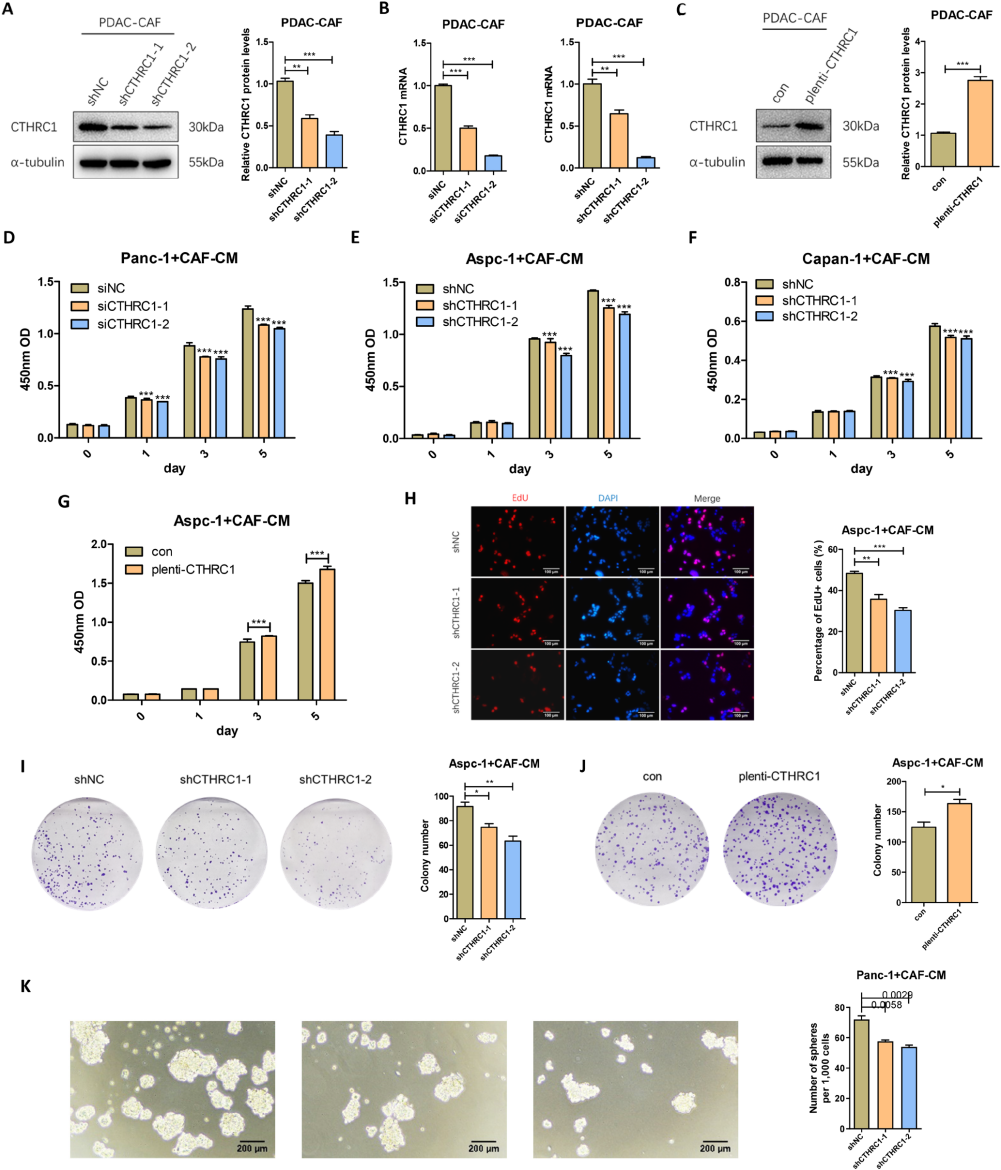
CTHRC1 in CAFs promotes proliferation and self‐renewal of pancreatic cancer cells in vitro. (A) Western blot detects the knockdown efficiency of CTHRC1 in CAFs and performs protein grayscale analysis. (B) CTHRC1 knockdown level in CAFs was measured by q‐PCR. (C) Western blot detects the overexpression level of CTHRC1 in CAFs and performs protein grayscale analysis. (D) Effect of CTHRC1 knockdown in CAFs on Panc‐1 proliferation. (E) Effect of CTHRC1 knockdown in CAFs on Aspc‐1 proliferation. (F) Effect of CTHRC1 knockdown in CAFs on Capan‐1 proliferation. (G) Effect of CTHRC1‐overexpressing CAFs on Aspc‐1 proliferation. (H) EdU fluorescence staining detects the effect of CTHRC1 knockdown in CAFs on Aspc‐1 proliferation (100 μm). (I) Effect of CTHRC1 knockdown in CAFs on the colony‐forming ability of Aspc‐1. (J) Effect of CTHRC1‐overexpressing CAFs on the colony‐forming ability of Aspc‐1. (K) Sphere formation assay detects the effect of CTHRC1 knockdown in CAFs on stemness‐related phenotypes in Panc‐1 cells. *p < 0.05; **p < 0.01; ***p < 0.001.

To explore whether CTHRC1 in pancreatic cancer CAFs has biological functions, we first constructed CAFs with CTHRC1 knockdown using siRNA transfection and lentiviral infection, and CAFs with CTHRC1 overexpression using lentiviral infection. The efficiency of CTHRC1 knockdown and overexpression was assessed by Western blot and q‐PCR. The results showed that both CTHRC1 protein and mRNA levels were significantly downregulated in CTHRC1 knockdown CAFs (Figure 2A,B), while CTHRC1 protein levels were significantly upregulated in CTHRC1 overexpressing CAFs (Figure 2C). These results indicate that we successfully established CAF cell models with CTHRC1 knockdown and overexpression, which can be used for subsequent experiments. We then investigated the role of CTHRC1 in CAFs in regulating pancreatic cancer cell proliferation. CAFs with CTHRC1 knockdown or overexpression were cocultured with pancreatic cancer cells, and the CCK‐8 cell proliferation assay showed that knockdown of CTHRC1 in CAFs significantly inhibited pancreatic cancer cell proliferation (Figure 2D–F), whereas overexpression of CTHRC1 in CAFs significantly promoted pancreatic cancer cell proliferation (Figure 2G). Additionally, EdU staining experiments demonstrated that knockdown of CTHRC1 in CAFs significantly inhibited pancreatic cancer cell proliferation and reduced cell numbers (Figure 2H). Similarly, colony formation assays also showed that CTHRC1 in CAFs significantly enhanced pancreatic cancer cell colony formation ability (Figure 2I,J). Furthermore, we conducted sphere formation assays to investigate the regulatory role of CTHRC1 in CAFs on tumor stemness potential. Compared with controls, CTHRC1‐knockdown CAFs significantly suppressed spherogenesis in Panc‐1 cells (Figure 2K), indicating that CAF‐derived CTHRC1 promotes self‐renewal capacity and stemness maintenance in pancreatic cancer cells. Thus, these findings demonstrate that CTHRC1 in CAFs substantially enhances the proliferative functions of pancreatic cancer cells and likely potentiates self‐renewal capabilities in PDAC cells.

### 3.3 CTHRC1 in CAFs Promotes the Migration and Invasion Functions of Pancreatic Cancer Cells In Vitro

**FIGURE 3:**
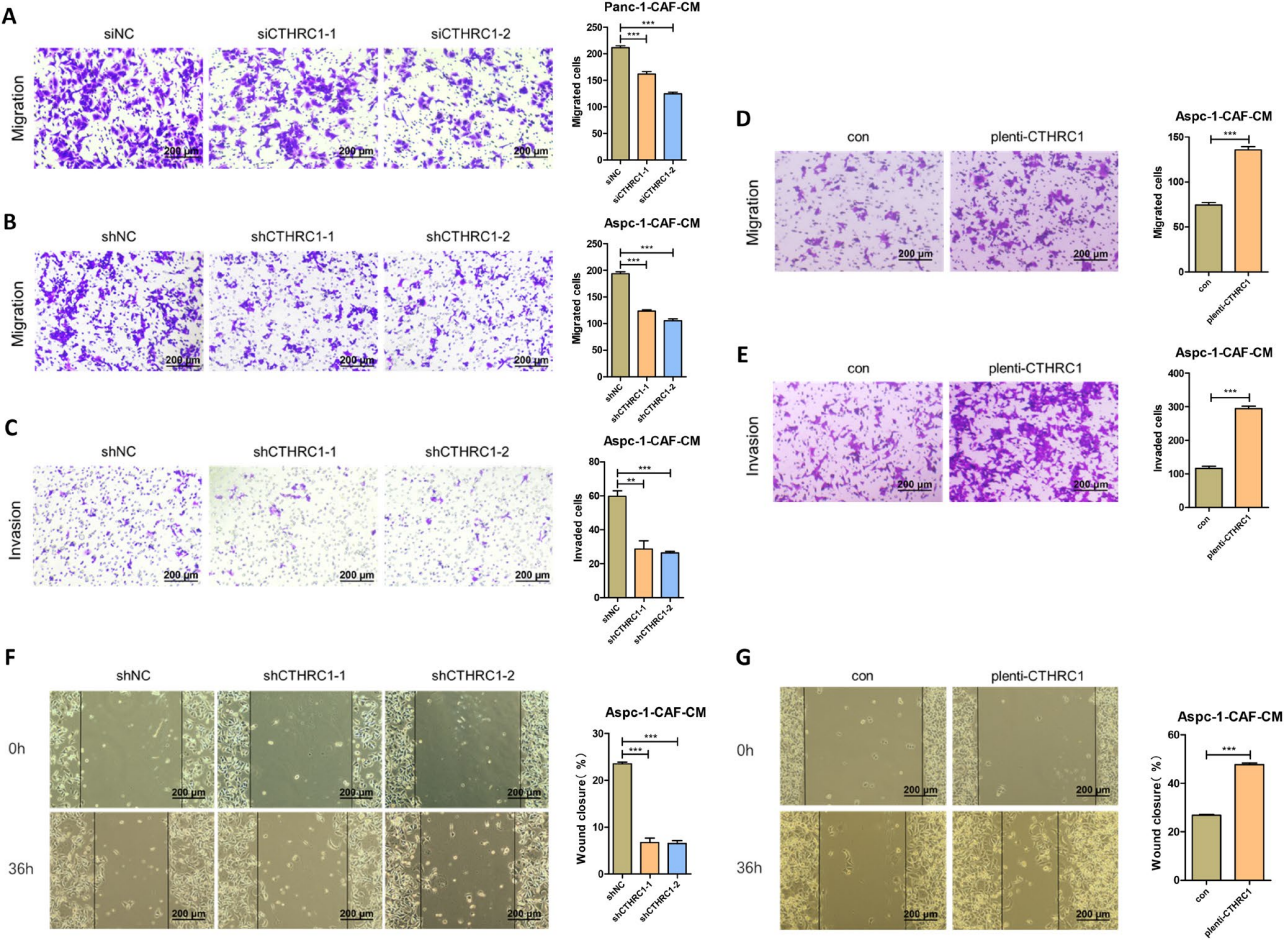
CTHRC1 in CAFs promotes the migration and invasion functions of pancreatic cancer cells in vitro. (A) Effect of CTHRC1 knockdown CAFs on the migration function of Panc‐1 (200 μm). (B) Effect of CTHRC1 knockdown CAFs on the migration function of Aspc‐1 (200 μm). (C) Effect of CTHRC1 knockdown CAFs on the invasion function of Aspc‐1 (200 μm). (D) Effect of CAFs overexpressing CTHRC1 on the migration function of Aspc‐1 (200 μm). (E) Effect of CAFs overexpressing CTHRC1 on the invasion function of Aspc‐1 (200 μm). (F) Effect of CTHRC1 knockdown CAFs on the wound healing function of Aspc‐1 (200 μm). (G) Effect of CAFs overexpressing CTHRC1 on the wound healing function of Aspc‐1 (200 μm). **p < 0.01; ***p < 0.001.

Secondly, we investigated the regulatory role of CTHRC1 in CAFs on the migration and invasion of pancreatic cancer cells. Using CAFs‐CM with CTHRC1 knockdown or overexpression as the lower chamber medium, the results of the Transwell migration and invasion assays showed that knockdown of CTHRC1 in CAFs significantly inhibited the migration and invasion of pancreatic cancer cells (Figure 3A–C), whereas overexpression of CTHRC1 in CAFs significantly enhanced the migration and invasion of pancreatic cancer cells (Figure 3D,E). Similarly, the scratch wound healing assay results also indicated that CTHRC1 in CAFs significantly promoted the migration and healing capacity of pancreatic cancer cells (Figure 3F,G). Therefore, these experimental results suggest that CTHRC1 in CAFs significantly enhances the migration and invasion of pancreatic cancer cells.

### 3.4 CTHRC1 in CAFs Promotes the Proliferation, Migration, and Invasion Functions of Pancreatic Cancer Cells In Vivo

**FIGURE 4:**
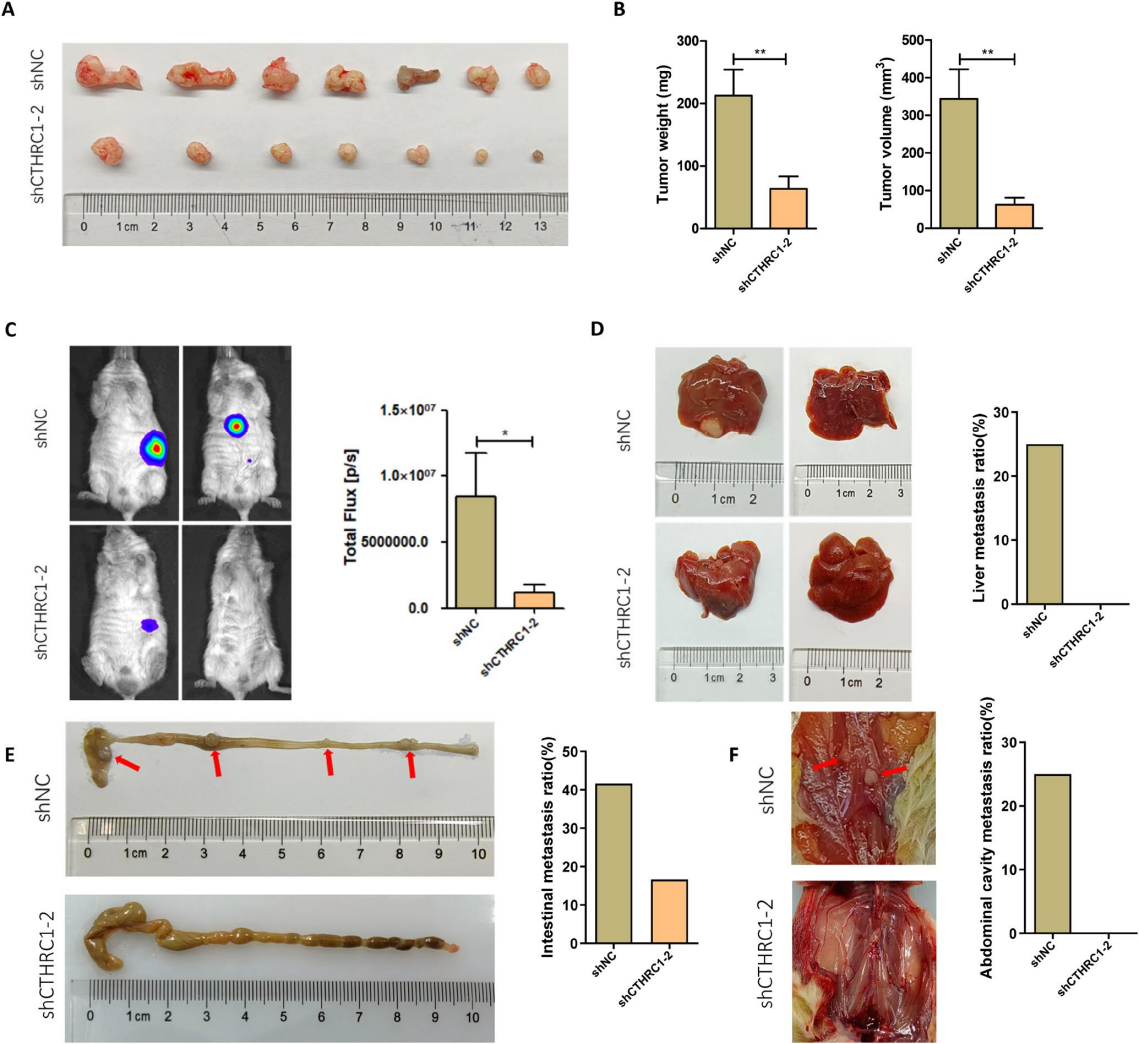
CTHRC1 in CAFs promotes the proliferation, migration, and invasion functions of pancreatic cancer cells in vivo. (A) Effect of CTHRC1 knockdown in CAFs on pancreatic cancer cell proliferation in vivo. (B) Analysis of tumor weight and volume in mice. (C) Representative images and quantitative analysis of in vivo imaging in mice. (D) Effect of CTHRC1 knockdown in CAFs on mouse liver metastasis and statistical analysis of the metastasis rate. (E) Effect of CTHRC1 knockdown in CAFs on mouse intestinal metastasis and statistical analysis of the metastasis rate. (F) Effect of CTHRC1 knockdown in CAFs on mouse peritoneal metastasis and statistical analysis of the metastasis rate. *p < 0.05; **p < 0.01.

In vitro experiments have confirmed that CTHRC1 in CAFs promotes the proliferation, migration, and invasion of pancreatic cancer cells. To further investigate the regulatory role of CTHRC1 in CAFs on pancreatic cancer progression in vivo, we established a mouse orthotopic pancreatic cancer xenograft model. CAFs with CTHRC1 knockdown and pancreatic cancer cells (Aspc‐1) were co‐injected into the mouse pancreas at a 1:3 ratio. After 8 weeks, in vivo imaging was performed to observe tumor growth and metastasis. The mice were then euthanized, and pancreatic tumors were excised. The in vivo results showed that, compared to the control group, CTHRC1 knockdown CAFs significantly inhibited the proliferation of pancreatic cancer cells (Figure 4A), and tumor volume and weight were significantly smaller than in the control group (Figure 4B). In vivo imaging further confirmed these results (Figure 4C). Additionally, CTHRC1 knockdown CAFs significantly inhibited the invasion and metastasis of pancreatic cancer to the liver, intestine, and peritoneal cavity compared to the control group (Figure 4D–F). Therefore, these animal experiments indicate that CTHRC1 in CAFs promotes the proliferation, migration, and invasion of pancreatic cancer cells in vivo.

### 3.5 CTHRC1 in CAFs Positively Regulates the Expression Level of LIF

**FIGURE 5:**
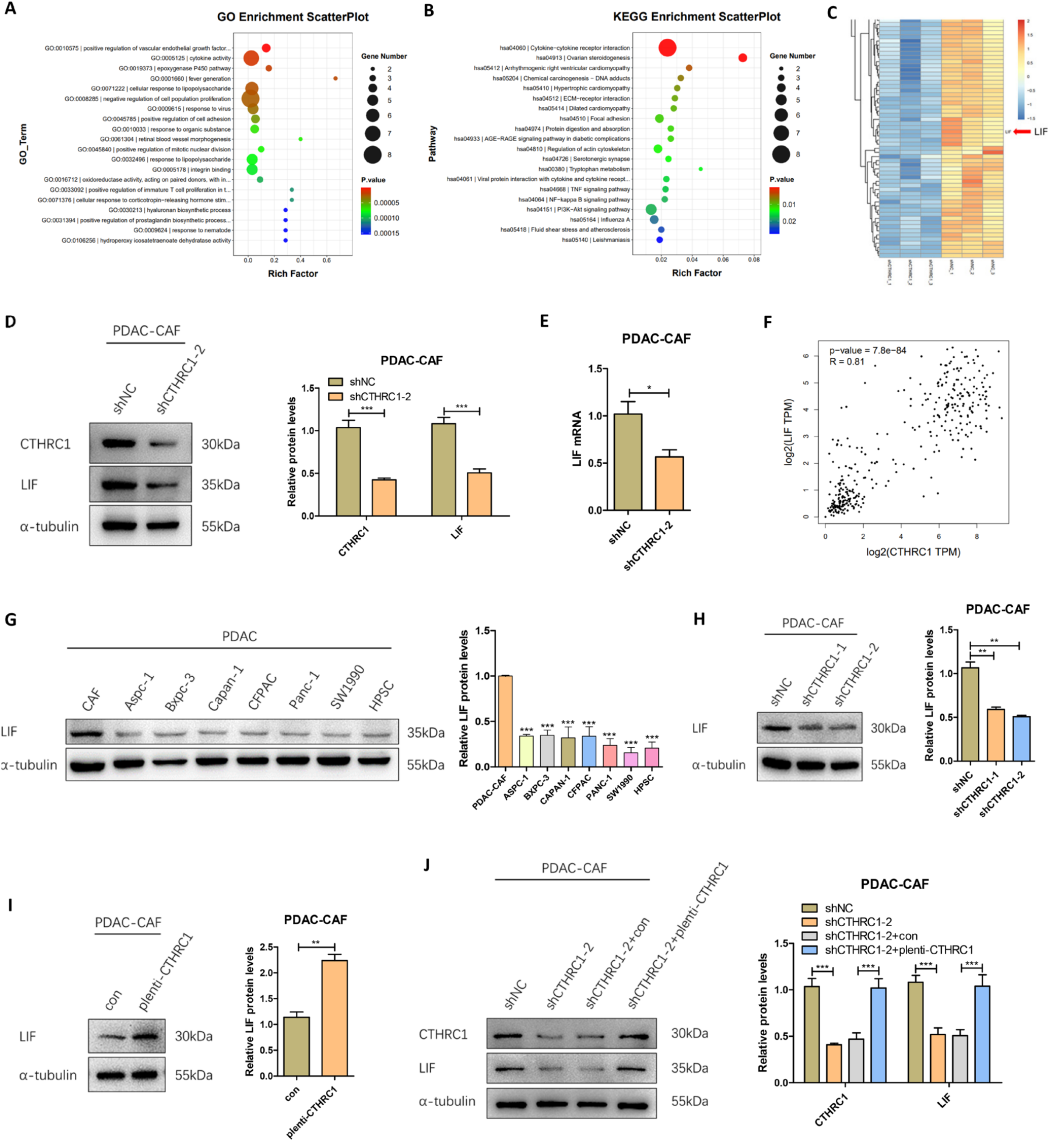
CTHRC1 in CAFs positively regulates the expression level of LIF. (A) RNA‐sequence was used to analyze the enrichment of differentially expressed genes regulated by CTHRC1 after knocking down CTHRC1 in CAFs. (B) RNA‐sequence was used to analyze the enrichment of gene signaling pathways regulated by CTHRC1. (C) Map of differentially expressed genes regulated by CTHRC1. (D) Western Blot was used to detect the expression of CTHRC1 and LIF in the sample of RNA‐sequence CAFs and protein gray scale analysis. (E) q‐PCR was used to detect the expression of LIF in the sample of RNA‐sequence CAFs. (F) Spearman correlation analysis combined with TCGA database was used to analyze the correlation between CTHRC1 and LIF. (G) Western Blot was used to detect the expression of LIF in CAFs, pancreatic cancer cell lines, HPSC, and protein gray scale analysis. (H) Western Blot was used to detect the expression of LIF in CTHRC1 knockdown CAFs and protein gray scale analysis. (I) Western Blot was used to detect the expression of LIF in CAFs overexpressing CTHRC1 and protein gray scale analysis. (J) Western Blot was used to detect the expression of CTHRC1 and LIF after the CAFs with CTHRC1 knockdown were reconstructed and overexpressed and protein gray scale analysis. *p < 0.05; **p < 0.01; ***p < 0.001.

Based on the in vitro and in vivo experiments described above, we have confirmed that CTHRC1 in CAFs significantly promotes the proliferation, migration, and invasion of pancreatic cancer cells, playing a crucial role in pancreatic cancer progression. To further explore the molecular mechanisms through which CTHRC1 exerts its effects in CAFs, we constructed stable shRNA knockdown cell lines in CAFs using lentiviral infection to knock down CTHRC1, and the knockdown efficiency was confirmed by Western Blot. We then performed RNA sequencing to analyze genes and signaling pathways that were upregulated or downregulated by more than twofold after CTHRC1 knockdown. The analysis revealed that the most significant changes in gene expression and enrichment were related to cytokines, and the cytokine‐cytokine receptor interaction pathway ranked first in the KEGG enrichment analysis (Figure 5A,B). Upon further examination of the molecular details, we found that LIF was one of the most significantly downregulated cytokines (Figure 5C). Western Blot and q‐PCR experiments on RNA‐Seq samples confirmed this result (Figure 5D,E). Subsequently, we interrogated the TCGA database again, and Spearman's analysis revealed a strong positive correlation between CTHRC1 and LIF (Figure 5F). Therefore, we decided to select LIF as the downstream target of CTHRC1. Furthermore, as a core effector of inflammatory CAFs [18], the CTHRC1‐LIF axis may become a potential therapeutic target for PDAC. To further clarify the regulatory role of CTHRC1 on LIF in CAFs, we first examined the differential expression of LIF in CAFs, pancreatic cancer cell lines, and human pancreatic stellate cells (HPSCs). We found that LIF expression was most significantly higher in CAFs, with very low expression in pancreatic cancer cells and HPSCs (Figure 5G). Next, we assessed LIF expression in CAFs after CTHRC1 knockdown and overexpression by Western Blot. The results showed that LIF expression was significantly downregulated after CTHRC1 knockdown (Figure 5H), and significantly upregulated after CTHRC1 overexpression (Figure 5I). Finally, we reconstituted CTHRC1 knockdown CAFs by overexpressing CTHRC1, extracted the cell proteins, and measured the expression levels of CTHRC1 and LIF by Western Blot. The results showed that after reconstitution, both CTHRC1 and LIF expression levels were restored (Figure 5J). Therefore, these experimental findings suggest that CTHRC1 in CAFs can positively regulate the expression of LIF.

### 3.6 CTHRC1 in CAFs Positively Regulates the Activation of STAT3 Pathway in Pancreatic Cancer Cells

**FIGURE 6:**
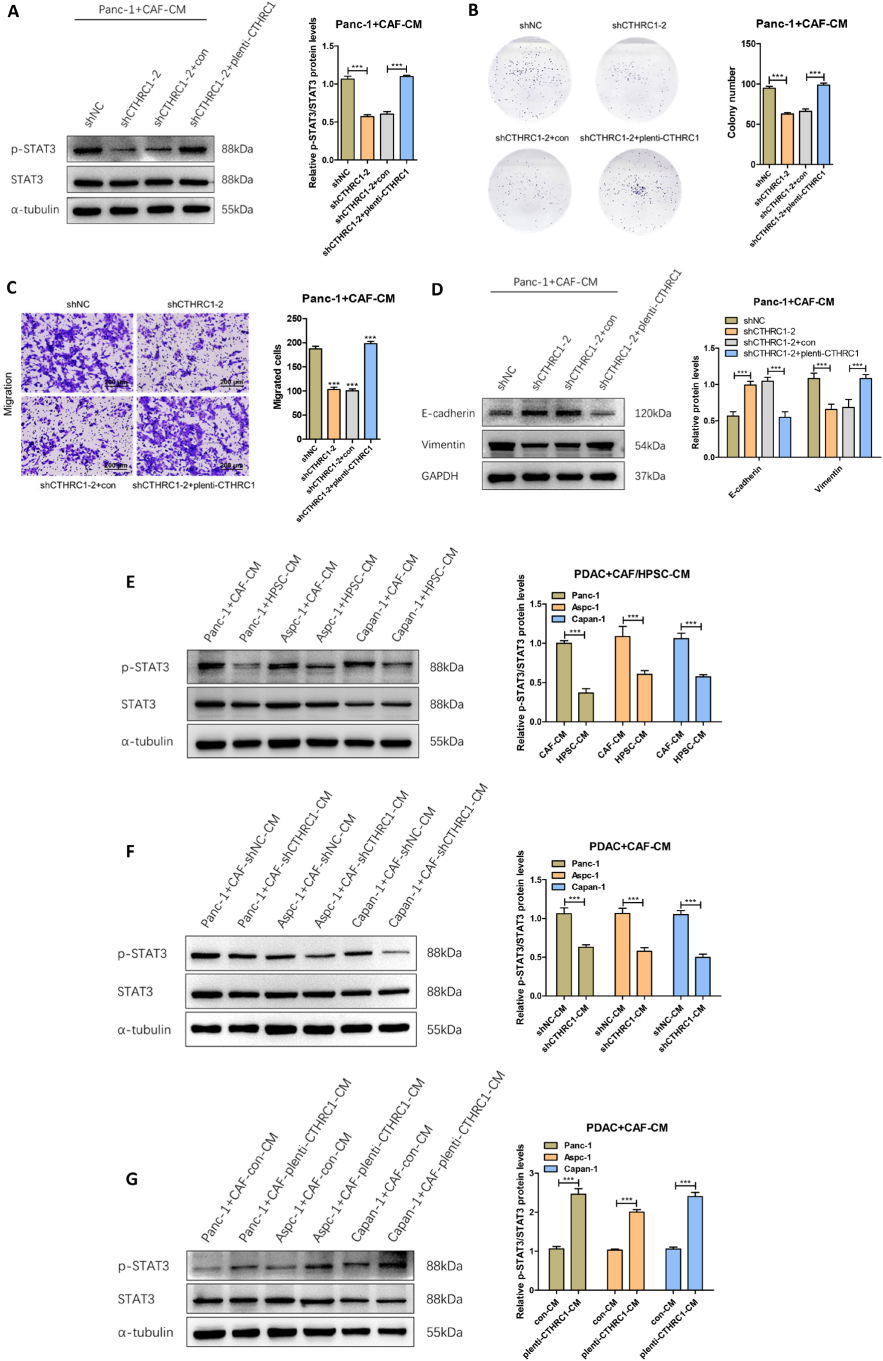
CTHRC1 in CAFs positively regulates the activation of STAT3 pathway in pancreatic cancer cells. (A) Western Blot was used to detect the activation of STAT3 signaling pathway after coculture of reconstructed CAFs‐CM with CTHRC1 overexpression and protein gray scale analysis. (B) Effect of CTHRC1 reconstructed and overexpressed CAFs on the colony‐forming ability of Panc‐1. (C) Effect of CTHRC1 reconstructed and overexpressed CAFs on the migration function of Panc‐1 (200 μm). (D) Western Blot was used to detect the expression of E‐cadherin and Vimentin after coculture of reconstructed CAFs‐CM with CTHRC1 overexpression and protein gray scale analysis. (E) Western Blot was used to detect the activation of STAT3 signaling pathway after coculture of HPSC‐CM and CAFs‐CM with Panc‐1, Aspc‐1, Capan‐1, and protein gray scale analysis. (F) Western Blot was used to detect the activation of STAT3 signaling pathway after coculture of CTHRC1 knockdown CAFs‐CM with Panc‐1, Aspc‐1, Capan‐1, and protein gray scale analysis. (G) Western Blot was used to detect the activation of STAT3 signaling pathway after coculture of CAFs‐CM overexpressing CTHRC1 with Panc‐1, Aspc‐1, Capan‐1, and protein gray scale analysis. ***p < 0.001.

We have confirmed that CTHRC1 in CAFs can positively regulate the expression level of LIF. To further explore the detailed molecular mechanism and signaling pathway by which CTHRC1 in CAFs regulates the progression of pancreatic cancer, we reviewed the literature and found that existing studies have confirmed that LIF mainly acts by activating the STAT3 signaling pathway. Moreover, LIF is the most important molecule in activating the STAT3 signaling pathway in pancreatic cancer [19]. Therefore, to further investigate and verify the role of CTHRC1 in CAFs in regulating LIF and whether CTHRC1 also regulates and activates the STAT3 signaling pathway in pancreatic cancer cells, CAFs‐CM with CTHRC1 knockdown and reconstructed CAFs‐CM with CTHRC1 overexpression were cocultured with Panc‐1. After 24 h, we extracted the cell proteins and detected the activation of the STAT3 signaling pathway using Western Blot. The results showed that knockdown of CTHRC1 in CAFs significantly downregulated the phosphorylation level of STAT3 in Panc‐1. After the CAFs with CTHRC1 knockdown were reconstructed and overexpressed, the upregulation of STAT3 phosphorylation in Panc‐1 was restored (Figure 6A). Additionally, we used clone formation and Transwell migration assays to explore the effects on proliferation and migration in pancreatic cancer cells after CAFs with CTHRC1 knockdown were reconstructed and overexpressed. The results showed that CTHRC1 knockdown in CAFs significantly inhibited the proliferation and migration of pancreatic cancer cells. However, after the CAFs with CTHRC1 knockdown were reconstructed and overexpressed, the promotion of proliferation and migration was restored (Figure 6B,C). Epithelial‐mesenchymal transition (EMT) plays a crucial role in tumor cell migration. We cocultured CAFs‐CM with Panc‐1 cells, extracted the cell proteins after 24 h, and detected the expression of EMT‐related markers E‐cadherin and Vimentin using Western Blot. The results showed that CTHRC1 knockdown in CAFs significantly upregulated the expression level of E‐cadherin and downregulated the expression level of Vimentin in Panc‐1. After the CAFs with CTHRC1 knockdown were reconstructed and overexpressed, the downregulation of E‐cadherin and upregulation of Vimentin expression in Panc‐1 were restored (Figure 6D). Therefore, we conclude that CTHRC1 in CAFs enhances the epithelial‐mesenchymal transition ability of pancreatic cancer cells. We then cocultured HPSC‐CM and CAFs‐CM with pancreatic cancer cells and, after 24 h, extracted the cell proteins to detect the activation of the STAT3 signaling pathway. The results showed that activated CAFs significantly promoted the phosphorylation of STAT3 in pancreatic cancer cells and induced activation of the STAT3 signaling pathway (Figure 6E), suggesting that this may be related to the high expression of CTHRC1 in CAFs. Subsequently, CAFs‐CM with CTHRC1 knockdown or overexpression were cocultured with pancreatic cancer cells, and after 24 h, we extracted the cell proteins to detect the activation of the STAT3 signaling pathway. The results showed that CTHRC1 knockdown in CAFs significantly downregulated the phosphorylation level of STAT3 and inhibited the activation of the STAT3 signaling pathway in pancreatic cancer cells (Figure 6F). On the other hand, CAFs overexpressing CTHRC1 significantly increased the phosphorylation level of STAT3 in pancreatic cancer cells and promoted the activation of the STAT3 signaling pathway (Figure 6G). Therefore, the above experimental results suggest that CTHRC1 in CAFs positively regulates the activation of the STAT3 signaling pathway in pancreatic cancer cells.

### 3.7 CTHRC1 in CAFs Mediates the Activation of STAT3 Signaling Pathway in Pancreatic Cancer Cells by Positively Regulating LIF

**FIGURE 7:**
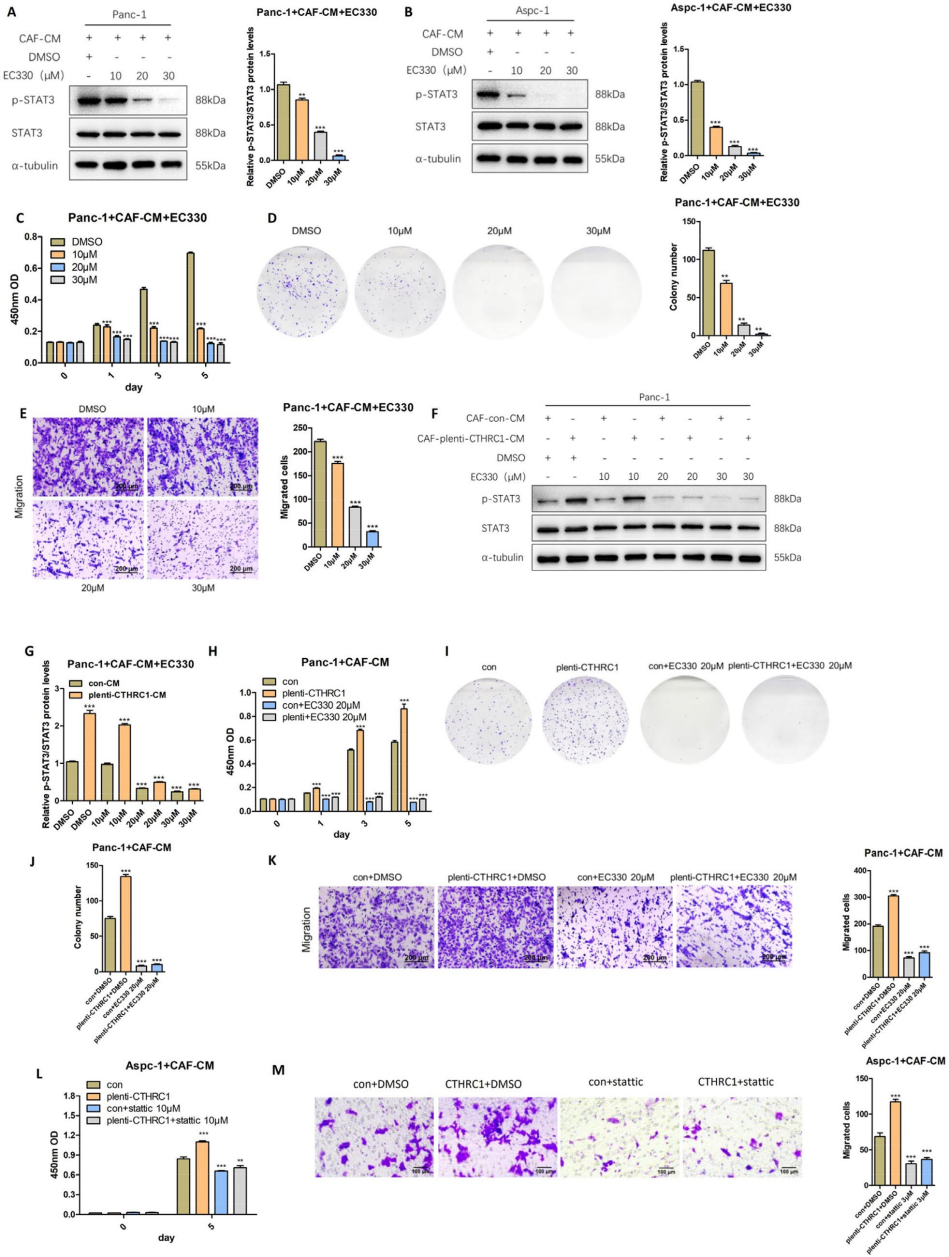
CTHRC1 in CAFs mediates the activation of STAT3 signaling pathway in pancreatic cancer cells by positively regulating LIF. (A) Western Blot was used to detect the activation of STAT3 signaling pathway in Panc‐1 after adding EC330 in CAFs‐CM and protein gray scale analysis. (B) Western Blot was used to detect the activation of STAT3 signaling pathway in Aspc‐1 after adding EC330 in CAFs‐CM and protein gray scale analysis. (C) Effect of CAFs‐CM on the proliferation function of Panc‐1 after adding EC330. (D) Effect of CAFs‐CM on the colony‐forming function of Panc‐1 after adding EC330. (E) Effect of CAFs‐CM on the migration function of Panc‐1 after adding EC330. (F, G) Western Blot was used to detect the activation of STAT3 signaling pathway in Panc‐1 after adding EC330 in CAFs‐CM overexpressing CTHRC1 and protein gray scale analysis. (H) Effect of CAFs‐CM overexpressing CTHRC1 on the proliferation function of Panc‐1 after adding EC330. (I, J) Effect of CAFs‐CM overexpressing CTHRC1 on the colony‐forming function of Panc‐1 after adding EC330. (K) Effect of CAFs‐CM overexpressing CTHRC1 on the migration function of Panc‐1 after adding EC330. (L) Effect of CAFs‐CM on the proliferative function of Aspc‐1 after adding Stattic. (M) Effect of CAFs‐CM overexpressing CTHRC1 on the migration function of Aspc‐1 after adding Stattic. **p < 0.01; ***p < 0.001.

Finally, to further explore and verify that CTHRC1 in CAFs activates the STAT3 signaling pathway in pancreatic cancer cells by regulating LIF, we cocultured CAFs‐CM with pancreatic cancer cells and added the LIF inhibitor EC330 at various concentrations. After 24 h, we extracted cell proteins and detected the activation of the STAT3 signaling pathway by Western Blot. The results showed that the phosphorylation level of STAT3 in pancreatic cancer cells was significantly downregulated after adding the LIF inhibitor, and the downregulation became more significant as the inhibitor concentration increased (Figure 7A,B). We also examined the effect of adding the LIF inhibitor to CAFs‐CM on the proliferation and migration of pancreatic cancer cells using CCK‐8, colony formation, and Transwell migration assays. The results showed that the proliferation and migration of pancreatic cancer cells were significantly inhibited after adding the LIF inhibitor, and the inhibitory effect was also significantly enhanced as the inhibitor concentration increased (Figure 7C–E). Then, we cocultured CAFs‐CM overexpressing CTHRC1 with pancreatic cancer cells and added the LIF inhibitor EC330 at various concentrations. After 24 h, we extracted cell proteins and detected the activation of the STAT3 signaling pathway by Western Blot. The results showed that the phosphorylation level of STAT3 in pancreatic cancer cells was significantly downregulated after adding the LIF inhibitor, and the downregulation became more significant as the inhibitor concentration increased (Figure 7F,G). Similarly, we also examined the effect of adding the LIF inhibitor to CAFs‐CM overexpressing CTHRC1 on the proliferation and migration of pancreatic cancer cells using CCK‐8, colony formation, and Transwell migration assays. The results showed that the proliferation and migration of pancreatic cancer cells were significantly inhibited after adding the LIF inhibitor, and the inhibition was significant as the inhibitor concentration increased (Figure 7H–K).

Finally, to further investigate whether CAF‐derived CTHRC1 activates the STAT3 signaling pathway in pancreatic cancer cells via LIF, we cocultured PDAC cells with conditioned medium from CTHRC1‐overexpressing CAFs (CAFs‐CM) in the presence of the STAT3‐specific inhibitor Stattic. CCK‐8 proliferation assays and Transwell migration assays were employed to examine the effects of STAT3 inhibition on pancreatic cancer cell proliferation and migratory capacity under CTHRC1‐overexpressing CAFs‐CM stimulation. Results demonstrated that Stattic treatment significantly suppressed both proliferative and migratory functions of pancreatic cancer cells (Figure 7L,M). These findings indicate that CTHRC1 in CAFs positively regulates the activation of the STAT3 signaling pathway in pancreatic cancer cells through LIF, thereby promoting their proliferation and migration.

## 4 Discussion

Pancreatic cancer is one of the deadliest cancers in the world. Early diagnosis is challenging, and most patients have lost the opportunity for surgery by the time it is detected, leading to an extremely poor prognosis. Even with the advancement of time and significantly improved medical technology, the 5‐year overall survival rate for pancreatic cancer patients remains only 11% [2]. Moreover, the incidence and mortality of pancreatic cancer continue to rise, with a trend toward younger patients [3]. Therefore, understanding the pathogenesis and progression of pancreatic cancer, and identifying new therapeutic strategies and targets, is crucial for its clinical diagnosis and treatment.

In recent years, a large number of studies have shown that the TME plays a crucial role in the occurrence and development, invasion, metastasis, and chemotherapy resistance of tumors. It is a dynamic and adaptive functional structure composed of tumor cells, stromal cells, immune cells, ECM, cytokines, and exosomes, which maintains the proliferation, invasion, and metastasis of tumor cells, forming a self‐perpetuating “ecosystem” for tumor advancement [7, 8]. Pancreatic cancer is rich in stroma, providing a natural adaptive medium for the proliferation, invasion, and metastasis of pancreatic cancer cells, as well as a stromal preparation for distant metastasis of pancreatic cancer [20], which is the result of the interaction between tumor cells and the TME. Additionally, the pancreatic cancer microenvironment has two main features: dense fibrous tissue proliferation and extensive immune suppression [21]. These two characteristics can promote the proliferation of pancreatic cancer cells by evading immune surveillance, either through direct inhibition of antitumor immunity or by inducing the proliferation and metastasis of immune suppressive cells [22]. This, in turn, promotes the metastasis and chemotherapy resistance of pancreatic cancer cells and enhances the communication between cancer cells and the stroma. Furthermore, it stimulates the secretion of ECM, vascular endothelial growth factor (VEGF), transforming growth factor‐β (TGF‐β), matrix metalloproteinases (MMPs), and other factors that maintain the TME [23, 24, 25]. This process continuously exacerbates pancreatic stromal fibrosis and immune suppression, thereby affecting the metabolism, proliferation, invasion, metastasis, and chemotherapy resistance of pancreatic cancer cells [26].

CAFs are an important component of the TME [23], and numerous studies have confirmed that they play a crucial role in tumor progression. They affect the remodeling of the tumor ECM, interact with cancer cells, regulate tumor cell proliferation, migration, and invasion, and contribute to chemotherapy resistance in cancer cells [27, 28, 29, 30]. In pancreatic cancer, CAFs have also been shown to be key factors in cancer progression. They are an integral part of the heterogeneous TME in pancreatic cancer and are involved in processes such as angiogenesis, lymphangiogenesis, epithelial‐mesenchymal transition, and the formation of the pre‐metastatic microenvironment [31, 32, 33]. Interestingly, CTHRC1+ CAFs exhibit significant functional plasticity across pathological contexts: in pulmonary fibrosis, they enhance tumor aggressiveness via the TGFβ1‐SFRP1 axis [25]; in lung cancer, they promote chemoresistance through TGF‐β/Smad3 signaling and a glycolysis/H3K18la positive feedback loop [34]; in pancreatic cancer, prior studies revealed spatial colocalization of CTHRC1+ CAFs with SPP1+ macrophages within PDAC microenvironments, where they collaboratively drive tumor progression by promoting fibrosis, immunosuppression, and epithelial‐mesenchymal transition [35]. Our current study demonstrates that in pancreatic cancer, CTHRC1+ CAFs primarily rely on the LIF/STAT3 signaling pathway to promote pancreatic cancer cell growth and metastasis. This context‐dependent functionality highlights microenvironmental signals as potential regulators of CTHRC1 activity. Notably, HPSCs—the primary cellular origin of pancreatic CAFs [36]—can be activated by platelet‐derived growth factor, TGF‐β, interleukins, and subsequently secrete pro‐tumorigenic factors including VEGF, LIF, and MMPs. The synergistic interplay between HPSCs and CTHRC1 drives tumor progression through ECM remodeling, metabolic reprogramming, and maintenance of immunosuppressive microenvironments [36, 37, 38, 39]. In‐depth elucidation of the microenvironment‐dependent mechanisms governing CTHRC1 will provide critical theoretical foundations for developing precision‐targeted therapeutic strategies.

This study, through single‐cell sequencing bioinformatics analysis [17], first identified CTHRC1 as specifically overexpressed in pancreatic cancer CAFs, with its expression significantly correlated to poor patient prognosis. Western blot, qPCR, and immunofluorescence experiments further confirmed CTHRC1 expression in CAFs substantially exceeds that in other cell types. Given the abundance of CAFs in the pancreatic cancer microenvironment, these findings indicate that CTHRC1 likely drives tumor progression. By establishing CTHRC1 gene‐edited CAF models (knockdown/overexpression) and applying their conditioned medium (CAFs‐CM) in cancer cell coculture systems, CCK‐8, colony formation, Transwell, and wound healing assays demonstrated CTHRC1 significantly promote cancer cell proliferation, migration, and invasion. In vivo experiments further validated its pro‐tumorigenic role, confirming CTHRC1 as a driver of pancreatic cancer progression and highlighting its potential as a therapeutic target.

To further clarify the detailed mechanism of CTHRC1 in CAFs in pancreatic cancer, we found that LIF may be one of the key downstream molecules of CTHRC1 in CAFs regulating pancreatic cancer progression by RNA‐sequence results and database analysis. LIF is an important component of the IL‐6 cytokine family [40]; studies have shown that the expression level of LIF in pancreatic cancer tissue is significantly increased compared with normal pancreatic tissue, and CAFs are the main cell source of LIF [41]. In pancreatic cancer, LIF secreted by CAFs binds to LIFR on the surface of pancreatic cancer cells and then activates the downstream STAT3 signaling pathway to play its biological role in promoting the progression of pancreatic cancer and chemoresistance [19, 42, 43]. We initially detected significantly higher LIF expression in CAFs compared to pancreatic cancer cell lines and HPSCs. Western blot analysis confirmed that CTHRC1 positively regulates LIF expression levels in CTHRC1‐knockdown, overexpressing, and reconstituted‐overexpressing CAF models. Subsequent coculture of pancreatic cancer cells with CTHRC1‐knockdown CAFs‐CM and CTHRC1‐reconstituted‐overexpressing CAFs‐CM demonstrated that CAF‐derived CTHRC1 promotes epithelial‐mesenchymal transition and positively regulates STAT3 signaling pathway activation in cancer cells. Colony formation and Transwell migration assays further revealed that genetic reconstitution of CTHRC1‐overexpressing CAFs restored their ability to promote pancreatic cancer cell proliferation and migration. Additional coculture experiments using HPSC‐CM, CTHRC1‐knockdown CAFs‐CM, and CTHRC1‐overexpressing CAFs‐CM consistently confirmed CTHRC1's positive regulation of STAT3 activation in pancreatic cancer cells. Notably, HPSC‐CM exhibited significantly weaker activation of the STAT3 signaling pathway compared to CAFs‐CM, indicating that CTHRC1 in CAFs plays a critical role in activating this pathway in pancreatic cancer cells. Finally, to further confirm that CTHRC1 in CAFs activates STAT3 signaling in pancreatic cancer cells via LIF regulation, we cocultured CTHRC1‐overexpressing CAFs‐CM with pancreatic cancer cells in the presence of the LIF inhibitor EC330 and STAT3 inhibitor Stattic. Results demonstrated that CTHRC1 in CAFs positively regulates LIF‐mediated STAT3 pathway activation. Furthermore, CCK‐8, colony formation, and Transwell migration assays revealed that CTHRC1 in CAFs promotes pancreatic cancer cell proliferation and migration through LIF‐dependent regulation.

Collectively, these in vitro and in vivo investigations confirm that CTHRC1 in CAFs upregulates LIF expression, thereby driving STAT3 pathway activation in pancreatic cancer cells and enhancing their proliferation and migration, positioning CTHRC1 as a potential diagnostic and therapeutic target in pancreatic cancer. However, the molecular mechanisms underlying CTHRC1‐mediated LIF regulation remain incompletely elucidated. Future studies will further dissect its specific roles in pancreatic cancer chemoresistance and targeted therapies, providing a theoretical foundation for precision therapeutic strategies.

## 5 Conclusions

**FIGURE 8:**
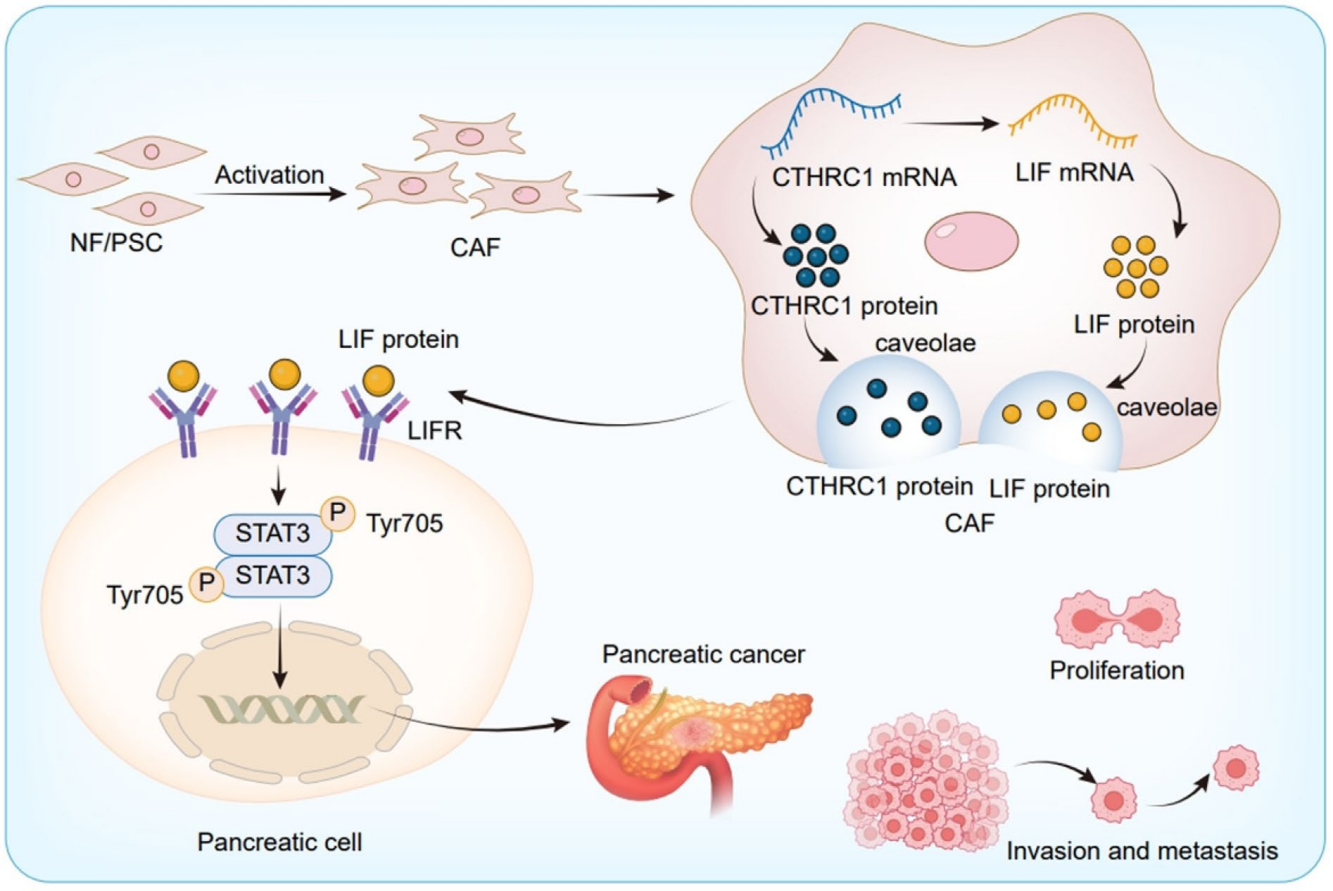
Schematic illustration of CAFs‐derived CTHRC1 promotes pancreatic cancer development and metastasis, cell migration, and tumor metastasis through the LIF‐STAT3 axis. CTHRC1 in CAFs can positively regulate the expression of LIF, thereby mediating the activation of the STAT3 signaling pathway in pancreatic cancer cells and promoting the proliferation and migration functions of pancreatic cancer cells.

In conclusion, CTHRC1 is highly expressed in CAFs of pancreatic cancer and is associated with the poor prognosis of patients. After knocking down CTHRC1 in CAFs, the proliferation, migration, invasion, tumor growth, and metastasis of pancreatic cancer cells were significantly inhibited. CTHRC1 in CAFs promotes the growth and metastasis of pancreatic cancer cells through the LIF/STAT3 signaling pathway (Figure 8). Therefore, our results reveal the role of CTHRC1 in CAFs in pancreatic cancer, and the CTHRC1/LIF/STAT3 signaling axis may be a promising prognostic marker and therapeutic target for patients with pancreatic cancer.

## Author Contributions

**Hang Yin:** conceptualization, investigation, methodology, validation, software, formal analysis, data curation, supervision, project administration, writing – review and editing, writing – original draft, visualization. **Yue Pan:** conceptualization, methodology, software, data curation, supervision, resources, formal analysis, project administration, visualization, validation, investigation, writing – original draft, writing – review and editing. **Zhuang Li:** methodology, validation, formal analysis, software, data curation, supervision. **Yong Liu:** conceptualization, methodology, software, data curation, supervision, formal analysis, validation, investigation. **Jiatong Chen:** conceptualization, methodology, software, data curation, supervision, investigation. **Xin Chen:** funding acquisition, visualization, project administration, resources, writing – review and editing. **Chengfei Zhang:** funding acquisition, visualization, project administration, resources, writing – review and editing. **Feng Zhu:** conceptualization, investigation, funding acquisition, visualization, validation, methodology, software, formal analysis, project administration, resources, supervision, data curation, writing – review and editing. **Chunzhao Yu:** funding acquisition, investigation, conceptualization, methodology, validation, visualization, project administration, formal analysis, software, data curation, supervision, resources, writing – review and editing.

## Ethics Statement

This study was approved by Ethics Review Committees/Institutional Review Boards of Sir Run Run Hospital Affiliated to Nanjing Medical University (Nanjing, China). All the animal experiments in this study were approved by the Institutional Animal Care and Use Committee (IACUC) of Nanjing Medical University (Nanjing, China). Clinical samples of pancreatic cancer and normal pancreatic tissue samples were all collected from Sir Run Run Hospital Affiliated to Nanjing Medical University. All patients involved in this article signed informed consent.

## Conflicts of Interest

The authors declare no conflicts of interest.

## Data Availability

Research data are stored in the repository of Sir Run Run Hospital Affiliated to Nanjing Medical University and will be shared upon request to the corresponding author.
